# The Role of Temporal Trends in Growing Networks

**DOI:** 10.1371/journal.pone.0156505

**Published:** 2016-08-03

**Authors:** Osnat Mokryn, Allon Wagner, Marcel Blattner, Eytan Ruppin, Yuval Shavitt

**Affiliations:** 1 Information and Knowledge Management Dept., University of Haifa, Haifa, Israel; 2 The Blavatnik School of Computer Science, Tel-Aviv University, Tel-Aviv, Israel; 3 Laboratory for Web Science, University of Applied Sciences (FFHS), & Tamedia Digital Analytics, Tamedia Zurich, Switzerland; 4 The Sackler School of Medicine, Tel-Aviv University, Tel-Aviv, Israel; 5 School of Electrical Engineering, Tel-Aviv University, Tel-Aviv, Israel; Georgia Institute of Technology, UNITED STATES

## Abstract

The rich get richer principle, manifested by the Preferential attachment (PA) mechanism, is widely considered one of the major factors in the growth of real-world networks. PA stipulates that popular nodes are bound to be more attractive than less popular nodes; for example, highly cited papers are more likely to garner further citations. However, it overlooks the transient nature of popularity, which is often governed by trends. Here, we show that in a wide range of real-world networks the recent popularity of a node, i.e., the extent by which it accumulated links recently, significantly influences its attractiveness and ability to accumulate further links. We proceed to model this observation with a natural extension to PA, named Trending Preferential Attachment (TPA), in which edges become less influential as they age. TPA quantitatively parametrizes a fundamental network property, namely the network’s tendency to trends. Through TPA, we find that real-world networks tend to be moderately to highly trendy. Networks are characterized by different susceptibilities to trends, which determine their structure to a large extent. Trendy networks display complex structural traits, such as modular community structure and degree-assortativity, occurring regularly in real-world networks. In summary, this work addresses an inherent trait of complex networks, which greatly affects their growth and structure, and develops a unified model to address its interaction with preferential attachment.

## Introduction

Many real world phenomena, and in particular self-organization properties observed in empirical data, have been explained as emergent properties of complex systems; the discipline of complex systems was applied to explain the structure of social and information networks, of computer networks, and of biological networks, to name a few [1]. One conspicuous example, which has been the subject of intense research [2–4], is the network of scientific collaboration. Previous studies considered various factors that might predict the future success of a paper to attract new citations. Among these are PA, also known as Price’s cumulative advantage model [2, 3, 5, 6]; a bias to cite recent papers [3, 7, 8] and relative fitness or significance each paper (or node) is born with, determined by importance and novelty [9, 10]; node similarity [11] and node aging, i.e. decaying attractiveness of older nodes [12–14]. However, none of these captures the phenomena of papers regaining popularity and becoming trending again.

**Fig 1:**
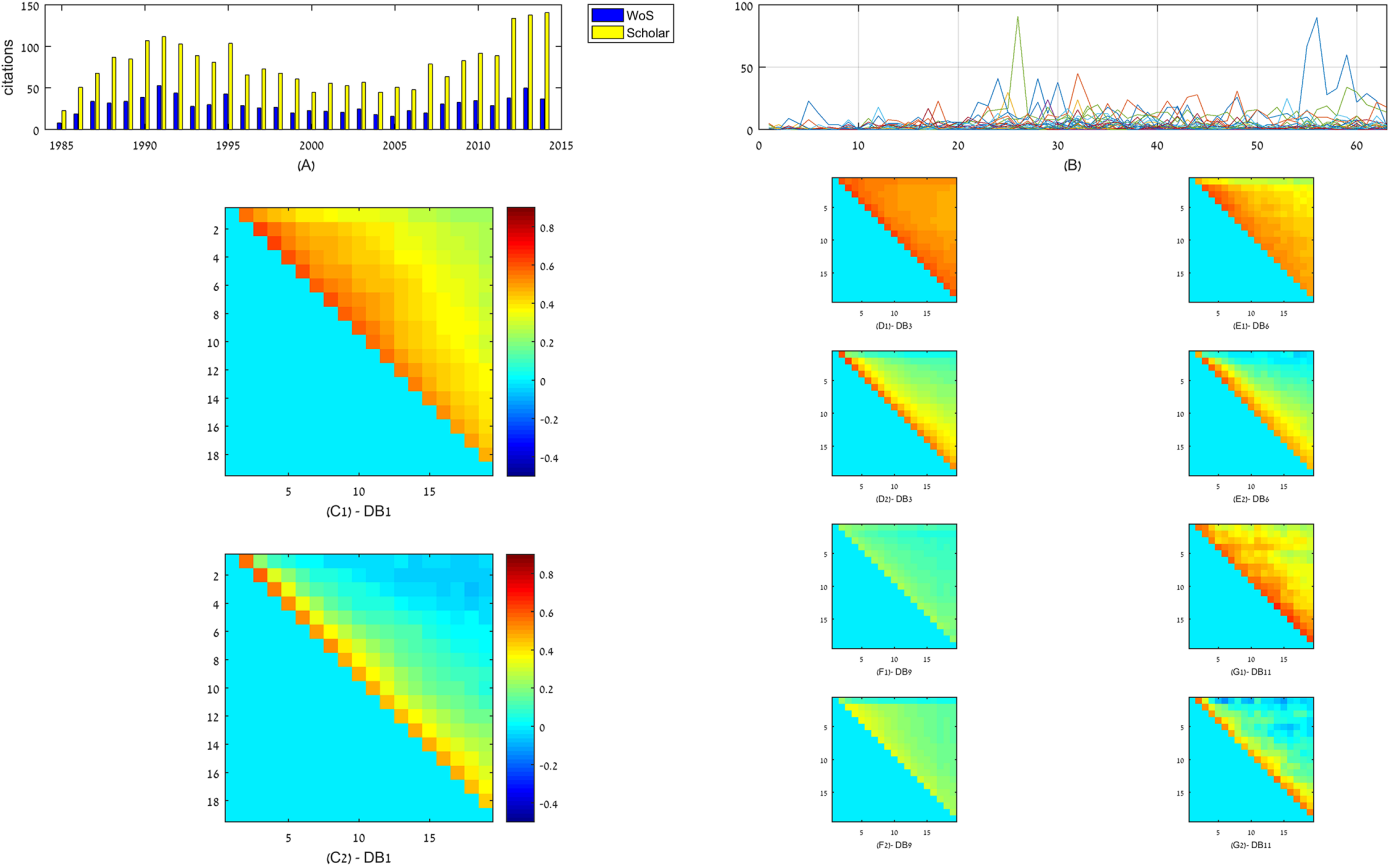
Trends in empirical data. (A-B) two illustrative cases of trend effects in networks. (A) Number of citations that a particular paper [24] garnered each year (according to Web of Knowledge–blue bars, or Google Scholar–red bars) gradually diminished after its publication, but then resurged due to renewed interest in its results. (B) Number of edits (y-axis) in 50 randomly chosen Wikipedia pages that were created on the same day shows fluctuating popularity. The x-axis represents 64 time bins of 60 days each. The number of edits per page changes with time with some of the pages accumulating links in several non-consecutive time intervals, in which their subject became trendy. (C-G) In real-world networks, links lose their relevance as they age. A pixel (*i*.*j*) in row *i* and column *j* such that *j* > *i* denotes the value of the Spearman correlation coefficient between the number of links each node accumulated in bins *i* and *j*. For each dataset, panel 1 gives the Spearman correlation, whereas panel 2 gives the partial Spearman correlation, controlled for the number of links a node had accumulated prior to bin *j* while excluding bin *i* (see main text). To allow comparison of networks with different time-scales, link addition events in which network were divided into *B* = 20 bins by their rank-transformed timestamps. Five datasets are presented in 2 panels each: C = Receiving side of Facebook wall posts; D = The passive part of a bipartite network containing users listening events to bands from the music website Last.fm; E = Scientific citation network: Arxiv HEP-PH (high energy physics phenomenology) citation graph; F = The passive part of a bi-partite network containing product ratings from the Amazon online shopping website; G = users posting in a Facebook-like forum network. Refer to the Section S1 Datasets for full descriptions of the data.

Trends are a driving force in our lives. From economy to online engagement and popular research subjects, the emergence and the spread of trends govern numerous aspects of modern social behavior [15–19]. For example, trends determine, to a large extent, the popularity distribution of user-generated content on YouTube. Video’s popularity generally diminishes quickly [20], and yet a few trending ones have become globally known, e.g., the Korean clip “Gangnam Style”, reaching over a billion views only months after its release [21], and totaling almost two billion views at present. Trends can also revive an already-faded popularity, as is the case with the renewed perception of Apple products as fashionable in the last decade, which has been instrumental to the company’s resurgence as a dominant actor in the computing industry after a long period of decline [22]. Trends are also evident in the Autonomous Systems Internet infrastructure, with specific ASes, such as Level3, Gblx, and Sprint, regaining popularity in 2008 following a drop of popularity the previous year [23]. The same effect can be detected in citation networks [10]. For instance, consider the paper by Ackley et al. [24] that described a novel learning technique for neural networks, and was well cited in the decade following its publication Fig 1A. Its yearly citation count started declining in the late 1990s and took off more recently due to a renewed interest in its applications in deep learning. In collaboration networks, it was shown that Wikipedia pages tend to be heavily edited shortly after appearing in a news outlet [25, 26], and see also Fig 1B.

Therefore, due to trends, networks evolve in ways that are sometimes inconsistent with preferential attachment, as is evident from Fig 1A and 1B. Most importantly, while preferential attachment can only predict that the most popular nodes will remain such [2], empirical evidence demonstrates the possibility that a late-coming node will garner enough popularity to become a central hub within the network.

## Results

### Analysis

The examples discussed so far indicate that trends can have a significant effect on the growth and self-organization of complex networks, and that recent history is potentially prominent in determining the current trend of nodes in networks. In order to assess the extent of this effect, we collected temporal data of eleven networks of various kinds: social interactions in online websites, academic citations, product recommendation and endorsement, Internet’s topology and so forth. The datasets are summarily described in Table 1, and full details are given in Section S1 Datasets. To normalize the time-scales of the different network, we divided the link addition events of each network into *B* = 20 bins according to the rank-transformed timestamps (rather than the actual timestamps). This ensured a uniform distribution of the link addition events across the bins, apart from the last bin which we exclude in the following analyses to avoid end-of-measurement effects.

**Table 1:** The datasets used in this work. Further information and links are given at Section S1 Datasets.

#	Name	Short Description	Nodes	Links	Period(months)
1	FBS	Facebook wall posts - senders	46952	274,086	58
2	FBR	Facebook wall posts - receivers	46952	274,086	58
3	FMB	Interactions with bands - FM	174077	4413834	58
4	FMU	Users interactions - FM	992	4413834	55
5	FMS	Interactions with songs - FM	1084620	4413834	55
6	CPH	Arxiv HEP-PH	34546	421578	124
7	CTH	Arxiv HEP-TH	27770	352807	124
8	IAS	Internet Autonomous Systems	58144	26749028	181
9	AMP	Amazon products’ ratings	1230915	5838041	88
10	WKE	Wikipedia edits	8752	466773	122
11	TSU	Tore users posting	1899	59835	6

**Fig 2:**
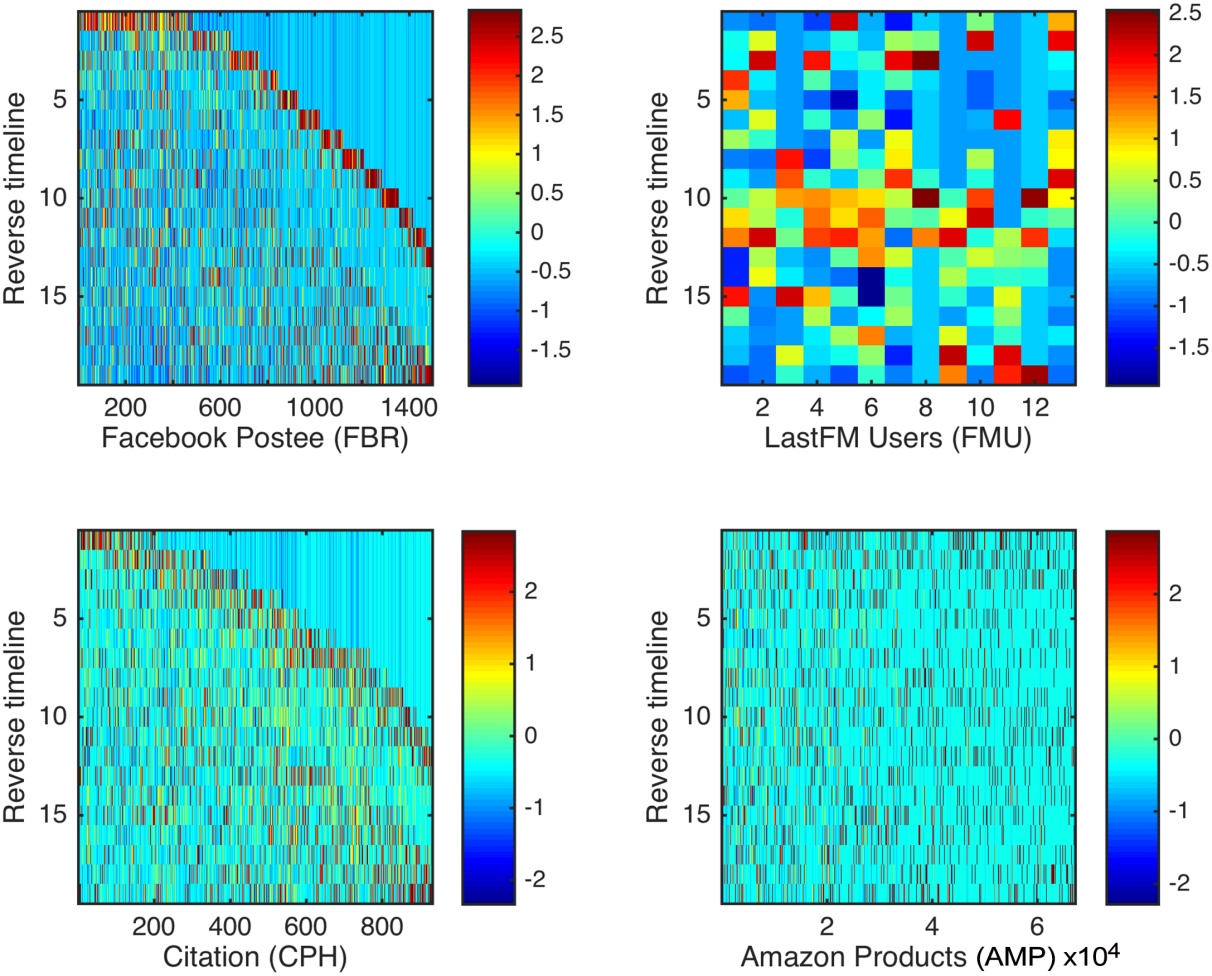
Reviving nodes popularity in empirical data. Nodes losing and regaining popularity in 4 networks from different domains. Each panel describes a subset of nodes (columns) of one network, with the y-axis corresponding to the time axis (top-to-bottom; time is divided into bins, see main text). The color intensity in pixel (*x*, *y*) represents the number of links node *x* had received in time bin *y*, z-normalized to allow presenting nodes with different degrees in a single color-scale. Nodes were chosen with a heuristic seeking nodes that had two separate time-points of peak popularity. It picked nodes where the two time bins in which the node accumulated the largest and second-largest number of links had similar scale, with a 10% difference at most, and were spaced at least 5 time bins apart. Additionally, at least in one of the separating time bins, the node had attracted not more than 10% of the number of links as in the second-largest bin. The number of nodes picked by the heuristic in each network, by the order of the panels, are 1498 (FBR); 13 (FMU); 932 (CPH); 67227 (AMP). See Table 1 for the size of each network.

We first verified that the datasets show evidence of nodes losing and then regaining popularity. Fig 2 shows this effect in 4 networks from different domains. Each panel shows a subset of such nodes (columns), and for each node shows the number of links it received in each of the time bins (rows). The nodes were detected with a simple heuristic that sought nodes for which the bins that contained the largest and second-largest numbers of link addition events were separated by the bins in which the node received far less links.

We then tested whether the number of links recently accumulated is predictive of the node’s future popularity, independently of PA effect. For each bin *i*, we considered the subset of nodes that were born by that time, denoted *S*_*i*_. Let *κ*_*v*_(*j*) denote the number of links a node *v* ∈ *S*_*i*_ accumulated in bin *j*. We computed the Spearman rank correlations between *κ*_*v*_(*i*)_*v* ∈ *S*_*i*__, the number of links these nodes had accumulated in bin *i*, and *κ*_*v*_(*j*)_*v* ∈ *S*_*i*__, the number of links these nodes had accumulated in any of the future bins *j* > *i*. As this metric is confounded by the popularity (namely, number of links) the node may have garnered in other bins, we also computed the partial correlation also while controlling for the number of links the node had accumulated in bins {1, …, *j* − 1} − {*i*}, namely, Σ_*x*=1,…,*i*−1,*i*+1,…,*j*−1_ *κ*_*v*_(*x*). Let *X*(*v*), *Y*(*v*), and *Z*(*v*) be random variables that denote the ranked number of links a node *v* accumulated in bins *i*, *j*, and {1, …, *j* − 1} − {*i*} (that is, without bin *i*), respectively. The partial correlation of *X* and *Y* across all nodes while controlling for Z is the correlation between the residuals *RX* and *RY* resulting from the linear regressions of *X* with *Z* and *Y* with *Z*, respectively. In our case *X*, *Y*, and *Z* are not the number of links a node had garnered, but rather the rank-transform of these random variables, which is necessary due to the non-linear effects in PA. Thus, the test is for monotonicity (Spearman) rather than linear correlation (Pearson). We observed a particularly strong correlation between the number of links in a time interval and in the time interval immediately preceding it; the correlation gradually declined when the latter was replaced with time intervals located in the more distant past (Fig 1C and 1D). (Correlations for all datasets appear in S1, S2 and S3 Figs). The results confirmed that the number of recently accumulated links is indeed predictive of the number of new links a node will accumulate, and that this effect is independent of PA, namely the predictive power of the node’s current degree.

### Trending preferential attachment

In light of the observations described above, we introduce a natural generalization to the widely accepted PA model [6], named Trendy Preferential Attachment (TPA). In PA, the probability of a new node to connect to a node *i* (denoted *ω*(*i*)) is proportional to the latter’s degree *k*_*i*_. Namely, *ω*(*i*) ∼ *k*_*i*_. We generalize PA by determining that the contribution of edges to the node’s appeal diminishes as a monotonically decreasing function *f*(*τ*) of their age *τ*. Thus, the probability of a new node to connect to a node *i* in time *t* is proportional to its time-weighted degree as follows: *ω*(*i*) ∼ *f*(1)*k*_*i*_(*t* − 1) + *f*(2)*k*_*i*_(*t* − 2) + ⋯ + *f*(*t* − 1)*k*_*v*_(1), with *k*_*i*_(*τ*) being the number of links that node *i* received in time *τ*. The function *f*(*τ*) captures the network’s trendiness, which may change between networks. We note that PA is a special case of TPA with *f*(*τ*) = 1 for every *τ*.

### Predictive capabilities of TPA

We tested whether TPA was better than PA at predicting the most popular nodes based on the network’s history. For that purpose, we assumed that in each of the empirical networks, link weights diminish as a polynomial function of the link’s age *τ* and an unknown parameter *α*, which we term the network’s *trending factor* (*f*(*τ*) ∼ 1/*τ*^*α*^,0 ≤ *α*, *α* = 0 corresponds to PA). For the datasets in this paper, *α* values smaller than 10 suffice. Given a real-world dataset to be modeled, we used a train set of the nodes to learn the parameter *α* through optimization, and then evaluated TPA’s performance on the rest of the nodes. For each network, link accumulation events were divided into *B* bins according to the rank of their respective timestamps. The last bin was excluded to avoid effects that are associated with end of network recording period. Nodes were divided into train sets and test sets in a 5-fold cross validation manner.

A simulated annealing optimization procedure was run in order to find *α* that minimized the *L*_1_ norm of the error vector. In detail, let *T* be the train set of nodes in a particular fold, and (as before) let *κ*_*v*_(*j*) denote the number of links a node *v* ∈ *S*_*i*_ accumulated in bin *j*. For each node *v* ∈ *T* we defined

$$\begin{array}{c} w_{v}^{B}\left(\alpha \right)=\Sigma_{i=1}^{B-1}\frac{1}{{\left(B-i\right)}^{\alpha }}\kappa_{v}\left(i\right) \end{array}$$

and

$$\begin{array}{c} d_{v}^{B}\left(\alpha \right)=\kappa_{v}\left(B\right)-\frac{w_{v}^{B}\left(\alpha \right)}{\Sigma_{u\in T}w_{u}^{B}\left(\alpha \right)}\Sigma_{u\in T}\kappa_{u}\left(B\right) \end{array}$$

The objective function to be minimized was then

$$\begin{array}{c} F^{B}\left(\alpha \right)=\frac{1}{N^{2}}{∥d_{v}^{B}\left(\alpha \right)∥}_{1} \end{array}$$

*N* denotes the number of nodes in the network; the multiplicative constant $\frac{1}{N^{2}}$ was added for normalization purposes and does not affect the optimization. In each fold, the optimal α* returned by the simulated annealing optimization procedure was used to compute the weights of each node *v* in the test set at the time of the last bin.

We evaluated $w_{v}^{B}\left(\alpha^{*}\right)$ on the test set by three criteria:

1. The Area Under the ROC Curve (AUC) of a predictor that relied on $w_{v}^{B}\left(\alpha^{*}\right)$ to predict the 20% nodes which accumulated the largest number of links at the last bin. i.e., nodes with the largest *κ*_*v*_(*B*).
2. The AUC of a predictor that relied on $w_{v}^{B}\left(\alpha^{*}\right)$ to predict the 20% nodes which accumulated the largest number of links at the last bin *relative to the number of links they have accumulated previously*. I.e., nodes with the largest $\kappa_{v}\left(B\right)/\left(\Sigma_{i=1}^{B-1}\kappa_{v}\left(i\right)\right)$. This evaluates the predictor’s quality at spotting nodes that might have relatively few links, but are growing fast and might turn into hubs.
3. The Kendall correlation coefficient between $w_{v}^{B}\left(\alpha^{*}\right)$ and *κ*_*v*_(*B*). The Kendall correlation reflects the number of concordant pairs vs. discordant node pairs. Let *u*, *v* be two nodes, such that $w_{v}^{B}\left(\alpha^{*}\right)>w_{u}^{B}\left(\alpha^{*}\right)$; the pair is concordant if *κ*_*v*_(*B*) > *κ*_*u*_(*B*) and discordant if *κ*_*v*_(*B*) < *κ*_*u*_(*B*). Whereas the first two criteria focus on the most popular nodes, this criterion measures the predictor’s quality at predicting the growth of all the nodes, rather than only the top growing ones.

In all cases, we report the average AUCs and average Kendall correlations across the 5 folds. Similarly, the α* values we report are the average across the folds.

**Fig 3:**
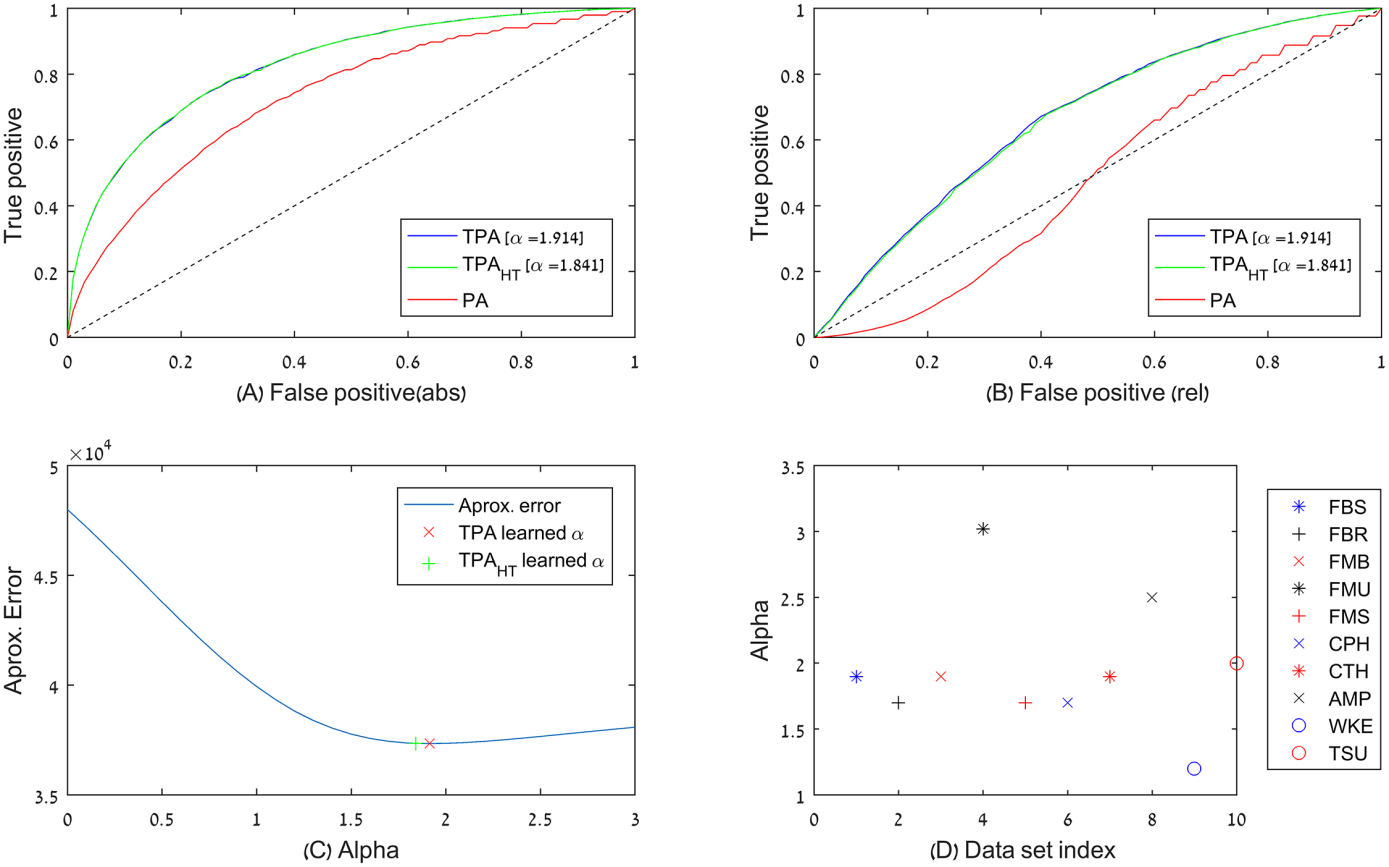
TPA vs. PA and trendiness of datasets. TPA predicts network growth better than PA. (A-C) show the results for one particular dataset (Facebook wall posts), while (D) summarizes the results of all the datasets used in this study. To normalize the time axes of the different datasets, link accumulation events in each network were divided into *B* = 20 by their rank-transformed timestamps. (A) ROC curve for TPA and TPA_HT_ (HT = half-time, refers to a TPA model trained only on the first half of the time axis, see main text), using the estimated *α* for each, when predicting the 20% nodes that accumulated most links. (B) The same, for predicting the top 20% nodes that accumulated most links relative to their previous degree. (C) The value of the error function used in the simulated annealing process (computed for all the nodes in the network) as a function of *α*, denoting the values to which TPA and TPA_HT_ converged (average of 5 folds for each). (D) For each of the 11 datasets, we estimate its learned *α* as described above, all showing notable trendiness. The IAS dataset with *α* = 7.1 is not shown.

Fig 3 shows that TPA outperformed PA in all cases, and as expected, the difference was mostly pronounced in networks with clear trendy dynamics (larger *α* values). Results for the remaining datasets are in S4, S5 and S6 Figs. Notably, TPA outperformed PA with respect to the second criterion. This reflects PA’s tendency to miss the emergence of new popular nodes, since it tends by definition to predict that the most popular nodes will remain such [2]. TPA, in contrast, is able to identify nodes that have recently accumulated many links as ones that are becoming increasingly popular.

To assess the sensitivity of TPA’s *α* estimation, we repeated the process described above, while limiting the time frame available to the train set for the purpose of learning *α* to only the first half of the time axis. We denote the result as TPA-half-time (TPA_HT_). TPA_HT_ was evaluated, as were TPA and PA, by its ability to predict the most popular nodes in the last time interval. Remarkably, its success was similar to that of TPA (Fig 3), which had the entire time axis available for training. TPA_HT_ demonstrates that *α* can be reliably estimated based only on partial history of the network, and then used to predict its future growth. S7 Fig depicts a comparison of the models for the top 20% most growing nodes; S8 Fig for the top 20% most growing nodes, relative to their degree. The insensitivity of our results to the precise value of *α* further indicates that the mere introduction of link loss of relevance as a function of their age greatly improves the predictive power of PA.

Notably, all tested data sets are considerably trendy (Fig 3D). The Wikipedia edit collaboration network is the less trendy in our dataset, with *α* = 1.2, implying that the number of edits per page is influenced by trends, but not as much as phenomena measured in the other datasets. On the other end of the scale is the very trending Internet Autonomous Systems infrastructure network (with *α* = 7.1), which has a short memory span, and in which existing links quickly lose their relevance in determining future link addition events. In conclusion, in all of the tested networks, nodes’ growth relies more on recent history than on their overall degree, in a way that conforms better to TPA than to PA.

### Structural properties of TPA

The complex structure of real-world networks does not manifest itself only in degree-distribution. We study TPA’s ability to recreate them through simulations with size *N* = 10000 nodes, and varying values of *α*. In each simulation, we initialized a network seed by generating a clique of size *m*_0_ = 100 nodes. All edges in the clique were considered as if they were created at time *t* = *m*_0_. Then, in each time step *t* = *m* + 1, …, *N*, one node *v*_*t*_ was added and connected to randomly chosen *m* = *m*_0_ nodes from those created at previous time steps (without replacement). The probability of *v*_*t*_ to connect to each of the previously created nodes was governed by TPA, as described in the main text, and thus depended on *f*(*τ*).

#### Modularity

A hallmark property of empirical networks, which has been the subject of intense research, is their tendency to naturally split into clusters, or communities [27, 28]. An optimal modularity measure [29] $Q\approx \sum_{ij}(A_{ij}-\frac{d_{i}d_{j}}{2m})s_{i}s_{j}$, *Q* ∈ (0, 1), quantifies the significance of extracted communities or clusters. We calculate *Q* following the heuristic method based on modularity optimization [30]. We average the modularity over all network instances belonging to the same *α*.

**Fig 4:**
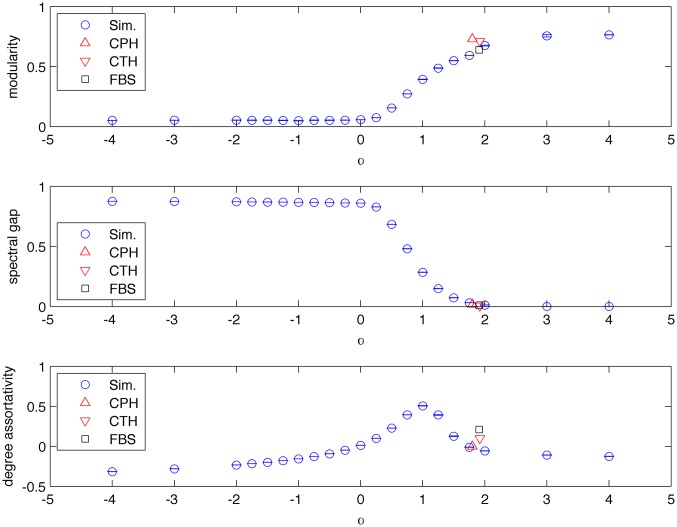
Structural properties of TPA networks. Modularity, spectral gap and degree-assortativity coefficient of TPA with link relevance that changes polynomial with their age (*f*(*τ*) ∼ 1/*τ*^*α*^). Each blue marker represents an average of 10 simulations. The special case *α* = 0 corresponds to a PA process. Red and black markers represent the computed values for 3 real-world networks of our datasets (the ones which were not bipartite): Facebook wall posts (FBS) and two sets of academic citations (CPH,CTH). *α* for the empirical networks was estimated through an optimization procedure, as described in the text.

We first examined the case of *alpha* ≪ 0, in which edges become more influential as they mature; we term such networks vintage networks. Fig 4 shows that for *alpha* ≪ 0 TPA generates a star like topology networks, not revealing any clear cluster partition. Vintage networks tend to show low modularity *Q* = 0. When *α* → 0 TPA generates PA-like networks, i.e., networks with modularity *Q* = 0. For 0 < *α* < 2 (moderately trendy networks) TPA generates more complex topologies, revealing clear community structures, with Q that increases monotonically as *α* increases. The networks maintain a high modularity, with Q eventually reaching a plateau, in highly trendy networks (*α* > 2).

#### Spectral gap

The TPA model generates an undirected and fully connected graph *G*(*V*, *E*), with *V* denoting the set of vertices and *E* the set of edges. We investigate the eigenvalue spectra and corresponding eigenvectors of the normalized Laplacian matrix *L*_*n*_(*A*) = *D*^−1/2^ *LD*^−1/2^, where *L* = *D* − *A* is the combinatorial Laplacian, *A*(*N* × *N*) is the adjacency matrix of the graph *G*, and *D* is the corresponding degree diagonal matrix. *L*_*n*_ is a positive semi-definite matrix with *N* real valued eigenvalues 0 ≤ *λ*_1_ ≤ *λ*_2_ ≤ ⋯*λ*_*N*−1_. It is well known that the eigenvalue spectra and the eigenvectors of *L*_*n*_ carry information about the graph topology [29, 31], e.g., community structure. For each network instance we use the implicitly restarted Lanczos method [32] to calculate the first 100 eigenvalues (increasing order) and the corresponding eigenvectors of the Laplacian matrix *L*_*n*_. We calculated the second eigenvalue of the Laplacian matrix of the corresponding network, as a function of *α*. Fig 4 indeed shows that as expected, the spectral-gap decreases when the modularity increases and vice versa [29]. TPA networks have markedly smaller spectral gaps than PA networks, and the spectral gap decreases as *α* increases. This again reflects the complex clustering structure of TPA networks, which also characterizes real-world networks, but is absent from PA-generated networks.

#### Assortativity

Another conspicuous property of real-world networks is the non-trivial assortativity of degrees, i.e. a tendency of high-degree nodes to prefer connecting (or avoid connecting) to other high-degree nodes [33]. In an assortative network highly connected nodes tend to connect to other highly connected nodes forming a core/periphery structure. Disassortative networks are characterized by star like structures, i.e., highly connected nodes tend to connect to low degree nodes. Both the Erd*ö*s-R*é*nyi and PA models produce, however, networks whose degree-assortativity tends to zero as their size tends to infinity. Fig 4 that TPA indeed gives rise to degree-assortative or disassortative networks. The degree assortativity coefficient was calculated by averaging over all instances belonging to the same *α*. When link attractiveness diminishes as a polynomial function of its age ($f\left(\tau \right)∼\frac{1}{\tau^{\alpha }},\alpha <1$), TPA networks are increasingly assortative, with a maximum at *α* = 1. At this point the assortativity coefficient decreases, and eventually becomes negative. Highly trendy networks (*α* > 1) are thus disassortative. Interestingly, vintage networks, for which *α* ≪ 0, are also disassortative. In summary, our simulations indicate that trending networks (0 < *α* < 2) show increasing modularity, decreasing spectral gap, and degree assortativity. Highly trendy networks (*α* > 2), on the other hand, are modular with a small spectral gap, while having a disassortative degree distribution. Vintage networks share this disassortative degree distribution, but show very low modularity.

#### Real world networks properties

Notably, the structural properties that TPA predicted matched those of real-world networks. We computed the modularity, spectral gap, and assortativity for a set of real world networks for which we have already computed the trending factor *α* (all the networks in our data that were not bipartite, namely, Facebook, and citation networks). Fig 4 shows that the values of these properties fit well with those of the TPA simulations with the same trending factors, as seen in Fig 4.

#### Further spectral properties

We further investigated spectral properties of TPA created networks for different values of the TPA network parameter, *α*. Namely, the spectrum of the normalized laplacian *L*_*n*_, and network projection into the Eigen space spanned by the first non-trivial eigenvectors of the laplacian matrix *L*_*n*_. These properties are further demonstrated for *α* ∈ [0..4] in S9–S18 Figs.

## Discussion

Previous studies have already considered various adaptations of the classic PA process [34] and explored its emergence in real-world networks [11]. However, there has been relatively little work on temporal effects. Lehmann et al. [35] observed that most papers are never cited beyond a particular point in time, and referred to them as “dead papers”. Consequently, they studied a PA process, in which nodes may die, and stop receiving links. Dorogovtsev and Mendes studied a PA model in which the probability of aged nodes to accumulate links is diminished [36]. Complementarily, papers may lose their appeal with time, as investigated in [12–14]. A long line of research has focused on a model in which nodes are assumed to have an intrinsic quality (called attractiveness, or fitness) that is combined with the usual rich-get-richer effect to determine their ability to draw new-coming edges. Nodes’ fitness can represent, for example, the inherent novelty and scientific rigor of a paper in a scientific citations network [9, 37, 38]. This inherent novelty, however, is assumed to be born with a node, and hence represents a model in which there is an extra parameter per node.

Here, we suggest that the tendency to adhere to trends is a network-wide property. Medo et al. [39] suggested that the fitness of nodes decreases with time, and this captures the timeliness of a paper. One notes that in all these studies a node can only lose its appeal with time. In contrast, our work offers a more general view of trend effects: a paper can not only become irrelevant and die, but also to gain renewed popularity. Our work demonstrates the existence of popularity momentums that feed on themselves and work in both directions: success is rewarded with further success, but failure increases the chance of further failure. In this respect, TPA addresses a problem stated already in 1976 by Price [5], who noted that PA models capture only the former, but not the latter, property. Consequently, TPA allows the emergence of new hubs, as well as the resurrection of old nodes, which naturally occur in real-world networks.

While we have limited the decay of a link’s importance in drawing more links to a power-law form (*f*(*τ*) ∼ 1/*τ*^*α*^), TPA can be generalized to accommodate any decay function, such as exponential decay on the one hand, or sub-polynomial decay on the other hand. We have further suggested that there may exist networks in which links become more influential as they age, i.e. *f*(*τ*) is monotonically increasing, which we have termed “vintage networks”.

## Supporting Information

S1 Datasets (PDF)

S1 Fig Temporal network analysis. This figure recapitulates Fig 1C and 1D for all the datasets analyzed in this paper. Link accumulation events were divided into 20 bins. A pixel (*i*, *j*) in row *i* and column *j* such that *j* > *i* denotes the value of the Spearman correlation coefficient between the number of links each node accumulated in bins *i* and *j*. For each dataset, panel 1 gives the Spearman correlation, whereas panel 2 gives the partial Spearman correlation, controlled for the number of links a node had accumulated prior to bin *j* while excluding bin *i*. Datasets: A. Facebook users receivers (FBR), B. Last-FM Bands (FMB), C. Last-FM Users (FMU).
(EPS)

S2 Fig Temporal network analysis. This figure recapitulates Fig 1C and 1D for all the datasets analyzed in this paper. Link accumulation events were divided into 20 bins. A pixel (*i*, *j*) in row *i* and column *j* such that *j* > *i* denotes the value of the Spearman correlation coefficient between the number of links each node accumulated in bins *i* and *j*. For each dataset, panel 1 gives the Spearman correlation, whereas panel 2 gives the partial Spearman correlation, controlled for the number of links a node had accumulated prior to bin *j* while excluding bin *i*. Datasets: A. Last-FM Songs (FMS), B. Academic citations: Arxiv HEP-TH (CTH), C. AS topology (IAS).
(EPS)

S3 Fig Temporal network analysis. This figure recapitulates Fig 1C and 1D for all the datasets analyzed in this paper. Link accumulation events were divided into 20 bins. A pixel (*i*, *j*) in row *i* and column *j* such that *j* > *i* denotes the value of the Spearman correlation coefficient between the number of links each node accumulated in bins *i* and *j*. For each dataset, panel 1 gives the Spearman correlation, whereas panel 2 gives the partial Spearman correlation, controlled for the number of links a node had accumulated prior to bin *j* while excluding bin *i*. Datasets: A. Amazon products ratings (AMP), B. Wikipedia edits (WKE), C. Tore Users (TSU).
(EPS)

S4 Fig TPA vs. PA and trendiness of datasets. For each dataset a panel with the following is given: ROC curve for TPA and *TPA*_*HT*_ (TPA that relies only on partial history), using the estimated *α* for each, when predicting the 20% nodes that accumulated most links; The same, for predicting the top 20% nodes that accumulated most links relative to their previous degree; The value of the error function used in the optimization process (computed for all the nodes in the network) as a function of *α*, denoting the values to which TPA and *TPA*_*HT*_ converged (average of 5 folds for each). First panel is for Facebook dataset (FBR) [A-C], Second panel is for Last_fm Bands (FMB) [D-F], and third is for Last_fm Users (FMU) [G-I].
(EPS)

S5 Fig TPA vs. PA and trendiness of datasets. For each dataset a panel with the following is given: ROC curve for TPA and *TPA*_*HT*_ (TPA that relies only on partial history), using the estimated *α* for each, when predicting the 20% nodes that accumulated most links; The same, for predicting the top 20% nodes that accumulated most links relative to their previous degree; The value of the error function used in the optimization process (computed for all the nodes in the network) as a function of *α*, denoting the values to which TPA and *TPA*_*HT*_ converged (average of 5 folds for each). First panel is for Last_fm Songs dataset (FMS) [A-C], Second panel is for Citations PH (CPH) [D-F], and third is for Citations TH (CTH)[G-I].
(EPS)

S6 Fig TPA vs. PA and trendiness of datasets. For each dataset a panel with the following is given: ROC curve for TPA and *TPA*_*HT*_ (TPA that relies only on partial history), using the estimated *α* for each, when predicting the 20% nodes that accumulated most links; The same, for predicting the top 20% nodes that accumulated most links relative to their previous degree; The value of the error function used in the optimization process (computed for all the nodes in the network) as a function of *α*, denoting the values to which TPA and *TPA*_*HT*_ converged (average of 5 folds for each). First panel is for Internet Autonomous Systems (IAS) Topology dataset [A-C]; Second panel is for Amazon products (AMP) [D-F]; Third is for Wikipedia edits (WKE) [G-I]; The last panel is for Tore social network users (TSU) [J-L].
(EPS)

S7 Fig Success of TPA, TPA_HT_, and PA in predicting the top 20% most growing nodes. For each of the 11 datasets, we show (left-to-right): AUC of TPA, TPA_HT_, PA in predicting the target 20% of the nodes; Kendall correlation between weights assigned by TPA, TPA_HT_, PA, and node growth (same in the two panels, duplicated for the reader’s convenience). Refer to the text for full details of their computation.
(EPS)

S8 Fig Success of TPA, TPA_HT_, and PA in predicting the top 20% most growing nodes, relative to their degree. For each of the 11 datasets, we show (left-to-right): AUC of TPA, TPA_HT_, PA in predicting the target 20% of the nodes; Kendall correlation between weights assigned by TPA, TPA_HT_, PA, and node growth (same in the two panels, duplicated for the reader’s convenience). Refer to the text for full details of their computation.
(TIF)

S9 Fig The PA Case. Spectrum and eigenprojection of the simulated network with *N* = 10000, *m* = 100, and *α* = 0.0. Colorbar indicates the normalized time. Blue dots represent old nodes, whereas red dots are younger nodes in the network. Top panel: spectrum of the normalized laplacian *L*_*n*_ and network projection into the eigenspace spanned by the first and second non-trivial eigenvector of the laplacian matrix *L*_*n*_. Bottom panel: projection into the eigenspace spanned by the first and third non-trivial eigenvector, respectively, vs. the second and third non-trivial eigenvector of the normalized laplacian matrix *L*_*n*_.
(TIF)

S10 Fig Spectrum and eigenprojection of the simulated network with *N* = 10000, *m* = 100, and *α* = 0.25. Color-bar indicates the normalized time. Blue dots represent old nodes, whereas red dots are younger nodes in the network. Top panel: spectrum of the normalized laplacian *L*_*n*_ and network projection into the eigenspace spanned by the first and second non-trivial eigenvector of the laplacian matrix *L*_*n*_. Bottom panel: projection into the eigenspace spanned by the first and third non-trivial eigenvector, respectively, vs. the second and third non-trivial eigenvector of the normalized laplacian matrix *L*_*n*_.
(TIF)

S11 Fig Spectrum and eigenprojection of the simulated network with *N* = 10000, *m* = 100, and *α* = 0.5. Colorbar indicates the normalized time. Blue dots represent old nodes, whereas red dots are younger nodes in the network. Top panel: spectrum of the normalized laplacian *L*_*n*_ and network projection into the eigenspace spanned by the first and second non-trivial eigenvector of the laplacian matrix *L*_*n*_. Bottom panel: projection into the eigenspace spanned by the first and third non-trivial eigenvector, respectively, vs. the second and third non-trivial eigenvector of the normalized laplacian matrix *L*_*n*_.
(TIF)

S12 Fig Spectrum and eigenprojection of the simulated network with *N* = 10000, *m* = 100, and *α* = 0.75. Colorbar indicates the normalized time. Blue dots represent old nodes, whereas red dots are younger nodes in the network. Top panel: spectrum of the normalized laplacian *L*_*n*_ and network projection into the eigenspace spanned by the first and second non-trivial eigenvector of the laplacian matrix *L*_*n*_. Bottom panel: projection into the eigenspace spanned by the first and third non-trivial eigenvector, respectively, vs. the second and third non-trivial eigenvector of the normalized laplacian matrix *L*_*n*_.
(TIF)

S13 Fig Spectrum and eigenprojection of the simulated network with *N* = 10000, *m* = 100, and *α* = 1.0. Colorbar indicates the normalized time. Blue dots represent old nodes, whereas red dots are younger nodes in the network. Top panel: spectrum of the normalized laplacian *L*_*n*_ and network projection into the eigenspace spanned by the first and second non-trivial eigenvector of the laplacian matrix *L*_*n*_. Bottom panel: projection into the eigenspace spanned by the first and third non-trivial eigenvector, respectively, vs. the second and third non-trivial eigenvector of the normalized laplacian matrix *L*_*n*_.
(TIF)

S14 Fig Spectrum and eigenprojection of the simulated network with *N* = 10000, *m* = 100, and *α* = 1.25. Colorbar indicates the normalized time. Blue dots represent old nodes, whereas red dots are younger nodes in the network. Top panel: spectrum of the normalized laplacian *L*_*n*_ and network projection into the eigenspace spanned by the first and second non-trivial eigenvector of the laplacian matrix *L*_*n*_. Bottom panel: projection into the eigenspace spanned by the first and third non-trivial eigenvector, respectively, vs. the second and third non-trivial eigenvector of the normalized laplacian matrix *L*_*n*_.
(TIF)

S15 Fig Spectrum and eigenprojection of the simulated network with *N* = 10000, *m* = 100, and *α* = 1.5. Colorbar indicates the normalized time. Blue dots represent old nodes, whereas red dots are younger nodes in the network. Top panel: spectrum of the normalized laplacian *L*_*n*_ and network projection into the eigenspace spanned by the first and second non-trivial eigenvector of the laplacian matrix *L*_*n*_. Bottom panel: projection into the eigenspace spanned by the first and third non-trivial eigenvector, respectively, vs. the second and third non-trivial eigenvector of the normalized laplacian matrix *L*_*n*_.
(TIF)

S16 Fig Spectrum and eigenprojection of the simulated network with *N* = 10000, *m* = 100, and *α* = 2.0. Colorbar indicates the normalized time. Blue dots represent old nodes, whereas red dots are younger nodes in the network. Top panel: spectrum of the normalized laplacian *L*_*n*_ and network projection into the eigenspace spanned by the first and second non-trivial eigenvector of the laplacian matrix *L*_*n*_. Bottom panel: projection into the eigenspace spanned by the first and third non-trivial eigenvector, respectively, vs. the second and third non-trivial eigenvector of the normalized laplacian matrix *L*_*n*_.
(TIF)

S17 Fig Spectrum and eigenprojection of the simulated network with *N* = 10000, *m* = 100, and *α* = 3.0. Colorbar indicates the normalized time. Blue dots represent old nodes, whereas red dots are younger nodes in the network. Top panel: spectrum of the normalized laplacian *L*_*n*_ and network projection into the eigenspace spanned by the first and second non-trivial eigenvector of the laplacian matrix *L*_*n*_. Bottom panel: projection into the eigenspace spanned by the first and third non-trivial eigenvector, respectively, vs. the second and third non-trivial eigenvector of the normalized laplacian matrix *L*_*n*_.
(TIF)

S18 Fig Spectrum and eigenprojection of the simulated network with *N* = 10000, *m* = 100, and *α* = 4.0. Colorbar indicates the normalized time. Blue dots represent old nodes, whereas red dots are younger nodes in the network. Top panel: spectrum of the normalized laplacian *L*_*n*_ and network projection into the eigenspace spanned by the first and second non-trivial eigenvector of the laplacian matrix *L*_*n*_. Bottom panel: projection into the eigenspace spanned by the first and third non-trivial eigenvector, respectively, vs. the second and third non-trivial eigenvector of the normalized laplacian matrix *L*_*n*_.
(TIF)
