# Supplementary material for: The Role of Temporal Trends in Growing Networks
Source: PLoS One. 2016 Aug 3;11(8):e0156505. doi: 10.1371/journal.pone.0156505 (PMC4972377; doi:10.1371/journal.pone.0156505)
Supplement: S1 Datasets — (PDF) [file pone.0156505.s001.pdf]

**S1 Datasets description** As link creation time is crucial for our research, we limited ourselves only to datasets temporal information on link creation was available. We obtained a large variety of datasets over several domains: technology, social, scientific, online product ratings, and a collaboration network. All data were accessed in September 2013, except for the Wikipedia data which was downloaded in July 2012. The datasets are referenced by the following codes in the text.

FBS A social interaction network, created by the group of users interacting through Facebook wall posts. A bi-partite network, of senders and receivers. The dataset was processed to obtain the sender side (posting users). The data covers posts written during the period of 2005 to 2009. Downloaded from *http://konect.uni-koblenz.de/networks/facebook-wosn-wall.*

FBR The receiver side of the social interaction bi-partite network, obtained by Facebook wall posts. This side of the network is the receivers - users whose wall received posts. The data covers posts written during the period of 2005 to 2009. Downloaded from *http://konect.uni-koblenz.de/networks/facebook-wosn-wall.*

FMB Obtained from a tripartite music social network from the "last.fm" music site. The network consists of bands, their songs, and the interactions of users. This part of the network is the "last-fm" Bands, and it constitutes the passive part of a bipartite network containing users listening events to bands from the music website "last.fm". The nodes are the bands listened to by users. The data was collected over the period of 2005 to 2009. Downloaded from *http://konect.uni-koblenz.de/networks/lastfm_band.*

FMU The social interaction between users in the Last-FM music site. The active part of a bipartite network containing users listening events to bands from the music website "last.fm". The nodes are the users listening to bands. The data was collected over the period of 2005 to 2009. Downloaded from *http://konect.uni-koblenz.de/networks/lastfm_band.*

FMS The passive part of the social music bipartite network, representing the users listening habits to songs of "last.fm" users. The nodes are the songs listened to by users. The data was collected over the period of 2005 to 2009. Downloaded from *http://konect.uni-koblenz.de/networks/lastfm_song.*

CPH Scientific citation network: Arxiv HEP-PH (high energy physics phenomenology ) citation graph [1, 2]. The data covers papers in the period from January 1993 to April 2003 (124 months). Downloaded from http://snap.stanford.edu/data/cit-HepPh.html. Additional processing was required for accumulating links per cited paper. We analyze link accumulation by the cited papers on a monthly basis.

CTH Scientific citation network: Arxiv HEP-TH (high energy physics theory) citation graph [1, 2]. The data covers papers in the period from January 1993 to April 2003 (124 months). Downloaded from: *http://snap.stanford.edu/data/cit-HepTh.html.* Additional processing was required for accumulating links per cited paper. We analyze link accumulation by the cited papers.

IAS A technology network of the Internet Autonomous systems (AS) topology. A graph describing the internet's AS-level topology between 1999 and 2013 [3], combining data from various sources. Data was accumulated and processed to identify the temporal information for link creation times. Downloaded from: *http://irl.cs.ucla.edu/topology*. Processed data will be given upon request.

AMP Amazon products. The passive part of a bi-partite network containing product ratings from the Amazon online shopping website. Nodes represent products. Edges represent individual ratings by the same users. The data covers papers in the period from January 1999 to April 2006. Downloaded from *http://konect.uni-koblenz.de/networks/amazon-ratings*.

WKE A collaboration network of Wikipedia edits. Wikipedia can be modeled as a collaboration network of its editors. Each article in Wikipedia is a result of collaboration of multiple authors (number of distinct authors for article has a median of 36.5 and follows a power-law [4]). The data contains Wiki pages, dates they were edited, and editor details. We then processed the data in the following manner. We modeled Wiki edits as a crowd-collaboration network, which is a network connecting users that collaborate on a project, without being directly connected. In a typical crowd-collaboration network the collaborators are modeled as the nodes, and links between them are created if they have collaborated on the same page. However, to account for the temporal nature of our research, we modeled the temporal behavior of a network as a stream of events. Each event in the stream represents a new link that was formed in the network and has few defining properties, such as the nodes it connect, and the creation timestamp of the event (Creation of a new revision of a page were modeled as an event in the event-stream). Timestamps are relative to the page creation time. This process allows us to analyze temporal aspects of activity: aggregating by period, comparing periods and looking for bursts of link creation. The edits were aggregated and binned over timescales of months, weeks, days and minutes. The data presented in this work is of weekly binning, for all Wikipedia pages that were created on a certain day. We selected February 25th 2002, in which a relatively large number of pages were created. Downloaded from: *http://dumps.wikimedia.org*.

TSU Tore_Users, an additional social network, which is a Facebook-like forum network. This is a bipartite graph (two-mode network) of users (first mode) posting to topics (second mode) in the same Facebook-like website as above [5]. We used the users information. The data covers the period between 14 May and 26 October 2004. Downloaded from: *http://toreopsahl.com/datasets*. In this dataset, we analyze the accumulation of links by users.

## References

1. Gehrke J, Ginsparg P, Kleinberg J. Overview of the 2003 KDD Cup. SIGKDD Explor Newsl. 2003;5(2):149–151.

2. Leskovec J, Kleinberg J, Faloutsos C. Graphs over time: densification laws, shrinking diameters and possible explanations. In: Proceedings of the eleventh ACM SIGKDD international conference on Knowledge discovery in data mining. ACM; 2005. p. 177–187.

3. Zhang B, Liu R. Collecting the Internet AS-level Topology. ACM SIGCOMM Computer Communications Review. 2005;35:53–61.

4. Voss J. Measuring Wikipedia. In: International Conference of the International Society for Scientometrics and Informetrics : 10th; July, 2005.

5. Opsahl T. Triadic closure in two-mode networks: Redefining the global and local clustering coefficients. Social Networks. 2013 May;35(2):159–167.
