# Supplementary figures and images for: The Role of Temporal Trends in Growing Networks
Source: PLoS One. 2016 Aug 3;11(8):e0156505. doi: 10.1371/journal.pone.0156505 (PMC4972377; doi:10.1371/journal.pone.0156505)

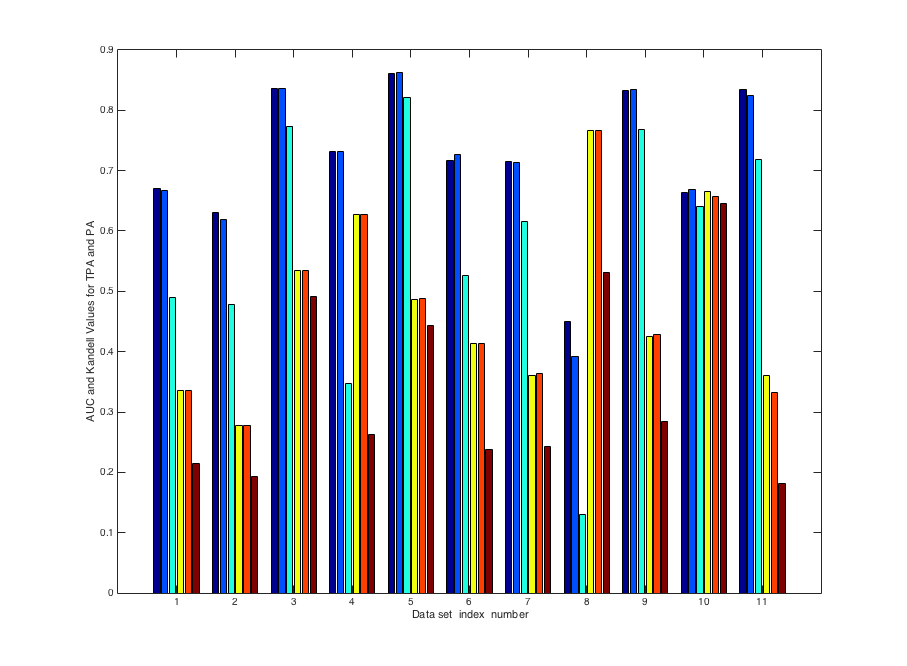

Supplement: S8 Fig — For each of the 11 datasets, we show (left-to-right): AUC of TPA, TPAHT, PA in predicting the target 20% of the nodes; Kendall correlation between weights assigned by TPA, TPAHT, PA, and node growth (same in the two panels, duplicated for the reader’s convenience). Refer to the text for full details of their computation. (TIF) [file pone.0156505.s009.tif]

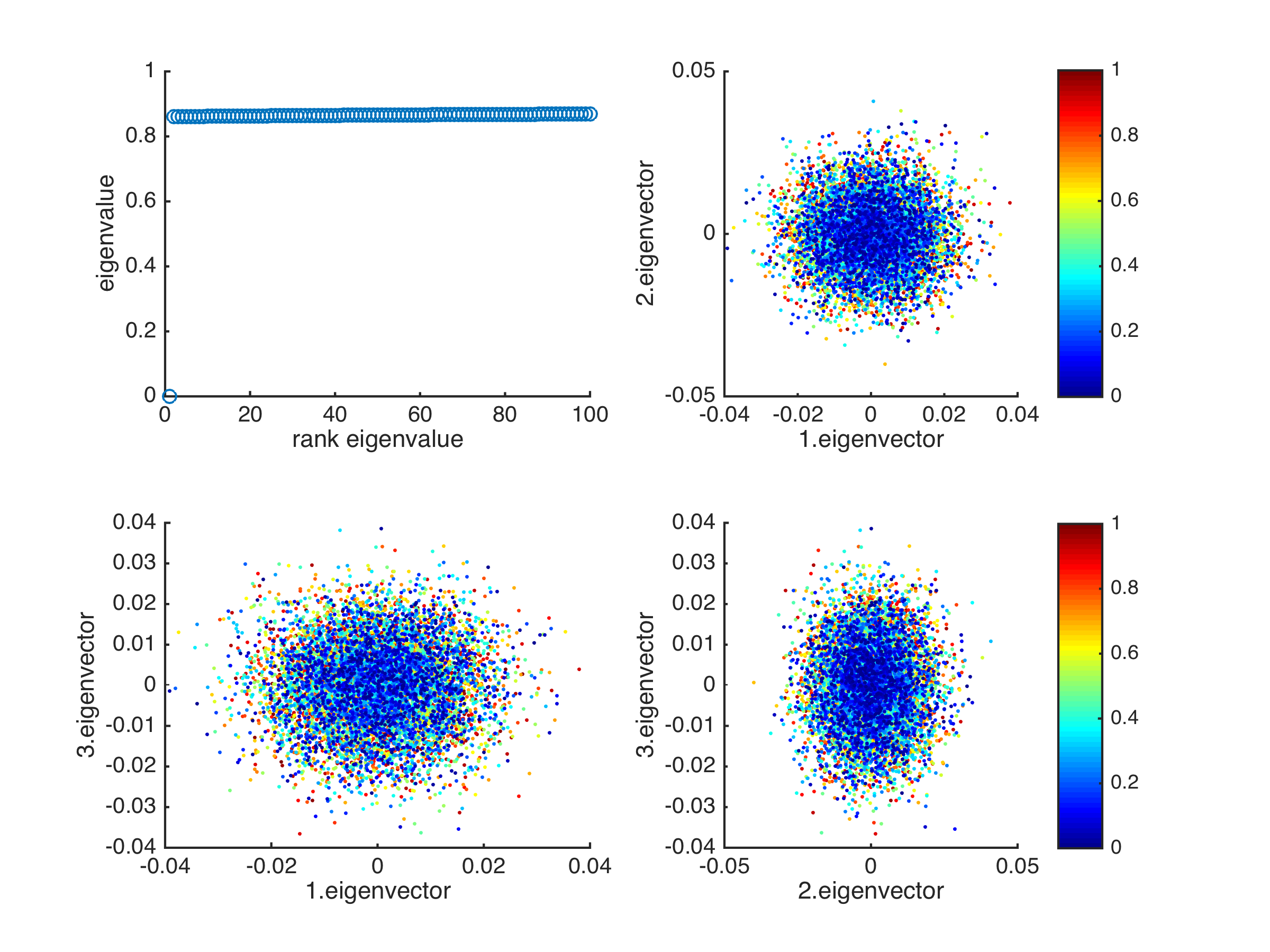

Supplement: S9 Fig — Colorbar indicates the normalized time. Blue dots represent old nodes, whereas red dots are younger nodes in the network. Top panel: spectrum of the normalized laplacian Ln and network projection into the eigenspace spanned by the first and second non-trivial eigenvector of the laplacian matrix Ln. Bottom panel: projection into the eigenspace spanned by the first and third non-trivial eigenvector, respectively, vs. the second and third non-trivial eigenvector of the normalized laplacian matrix Ln. (TIF) [file pone.0156505.s010.tif]

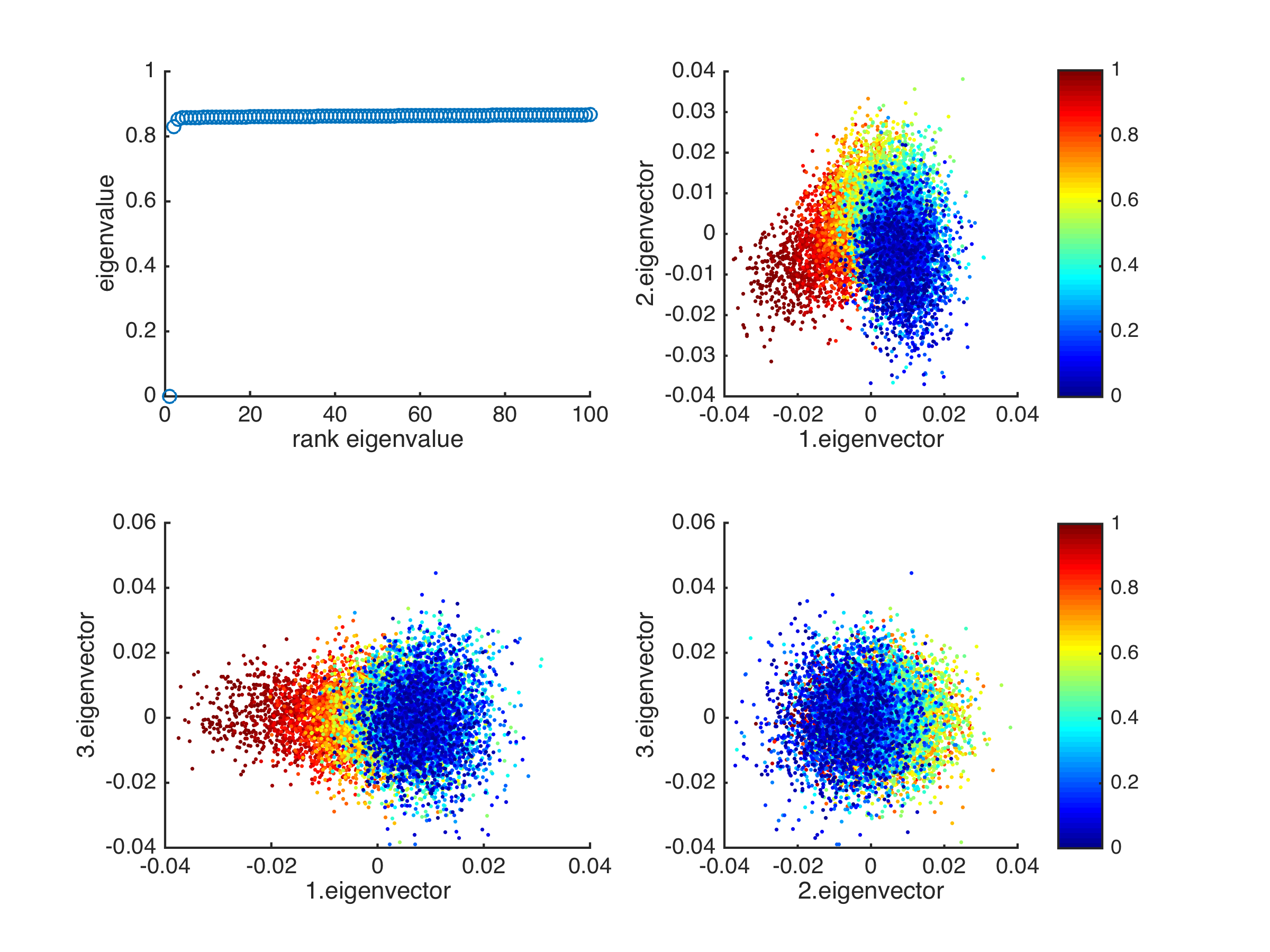

Supplement: S10 Fig — Color-bar indicates the normalized time. Blue dots represent old nodes, whereas red dots are younger nodes in the network. Top panel: spectrum of the normalized laplacian Ln and network projection into the eigenspace spanned by the first and second non-trivial eigenvector of the laplacian matrix Ln. Bottom panel: projection into the eigenspace spanned by the first and third non-trivial eigenvector, respectively, vs. the second and third non-trivial eigenvector of the normalized laplacian matrix Ln. (TIF) [file pone.0156505.s011.tif]

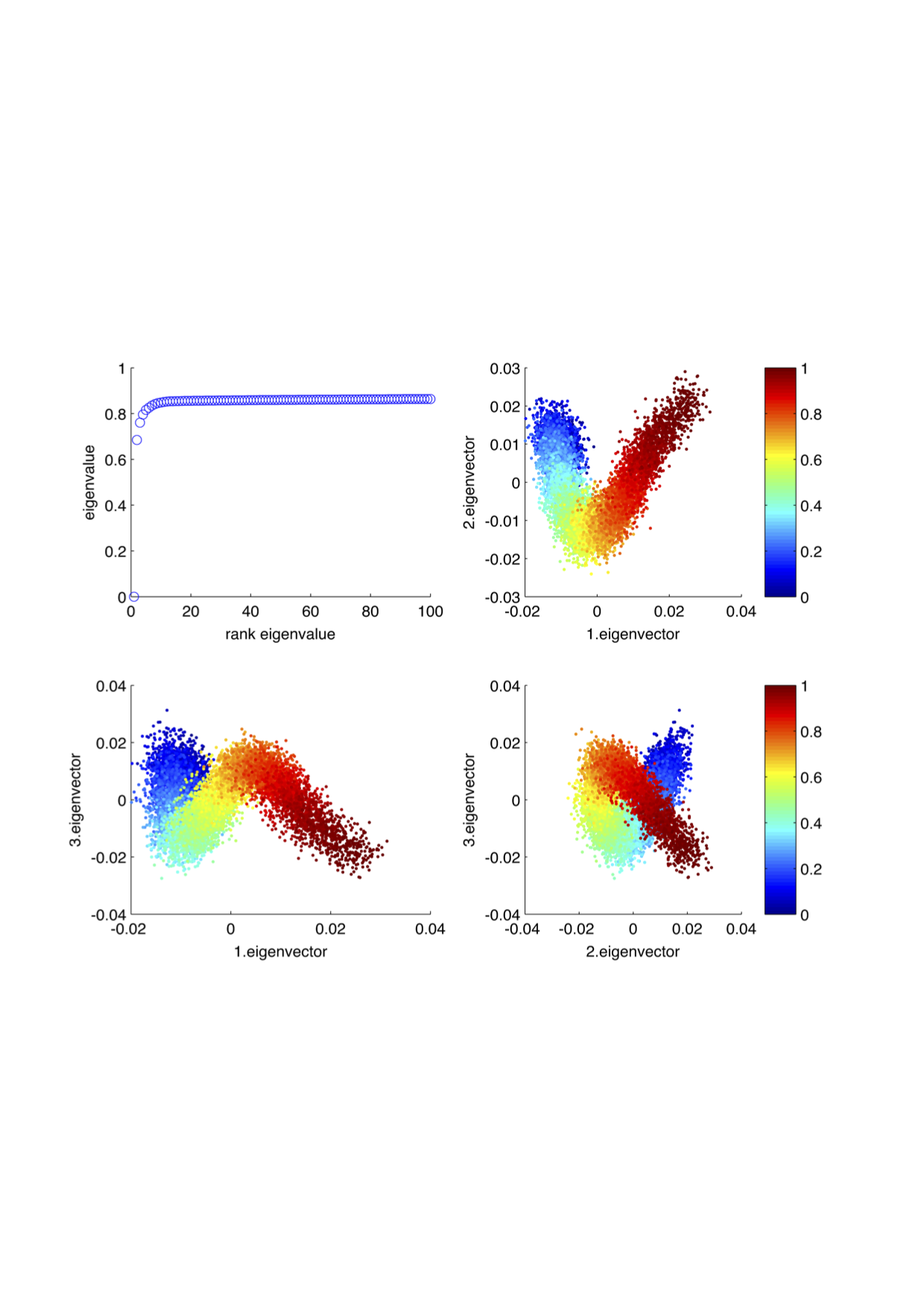

Supplement: S11 Fig — Colorbar indicates the normalized time. Blue dots represent old nodes, whereas red dots are younger nodes in the network. Top panel: spectrum of the normalized laplacian Ln and network projection into the eigenspace spanned by the first and second non-trivial eigenvector of the laplacian matrix Ln. Bottom panel: projection into the eigenspace spanned by the first and third non-trivial eigenvector, respectively, vs. the second and third non-trivial eigenvector of the normalized laplacian matrix Ln. (TIF) [file pone.0156505.s012.tif]

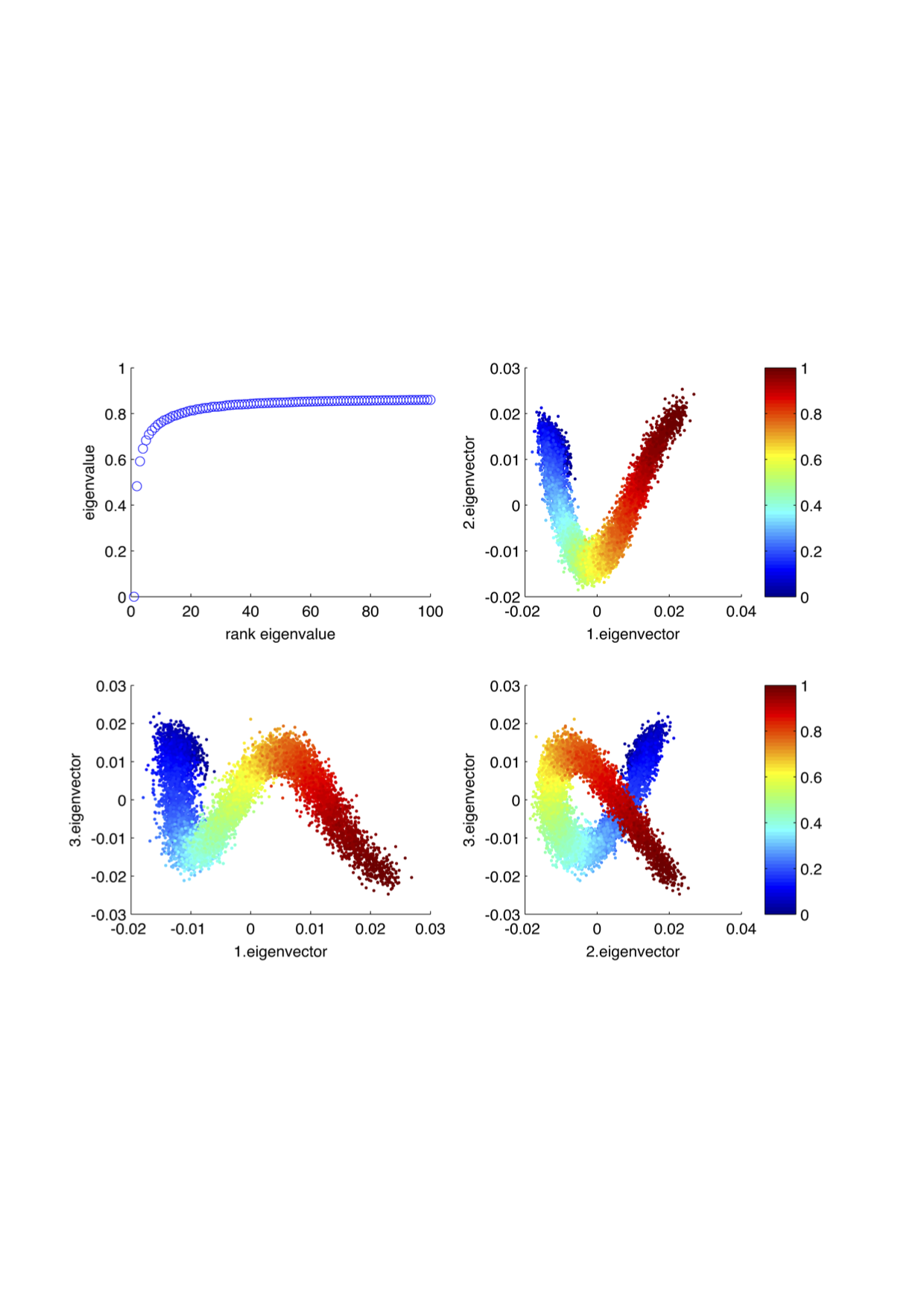

Supplement: S12 Fig — Colorbar indicates the normalized time. Blue dots represent old nodes, whereas red dots are younger nodes in the network. Top panel: spectrum of the normalized laplacian Ln and network projection into the eigenspace spanned by the first and second non-trivial eigenvector of the laplacian matrix Ln. Bottom panel: projection into the eigenspace spanned by the first and third non-trivial eigenvector, respectively, vs. the second and third non-trivial eigenvector of the normalized laplacian matrix Ln. (TIF) [file pone.0156505.s013.tif]

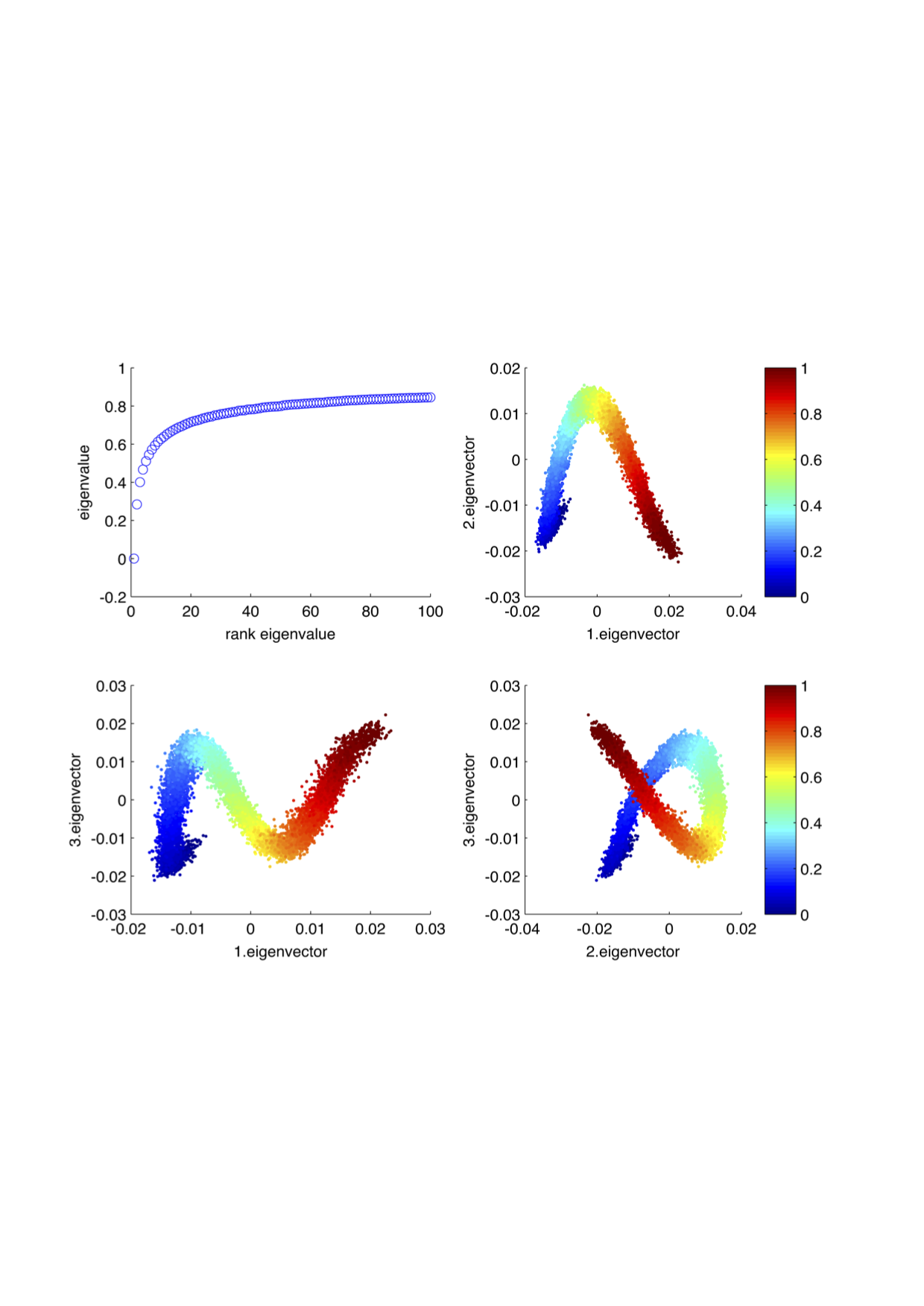

Supplement: S13 Fig — Colorbar indicates the normalized time. Blue dots represent old nodes, whereas red dots are younger nodes in the network. Top panel: spectrum of the normalized laplacian Ln and network projection into the eigenspace spanned by the first and second non-trivial eigenvector of the laplacian matrix Ln. Bottom panel: projection into the eigenspace spanned by the first and third non-trivial eigenvector, respectively, vs. the second and third non-trivial eigenvector of the normalized laplacian matrix Ln. (TIF) [file pone.0156505.s014.tif]

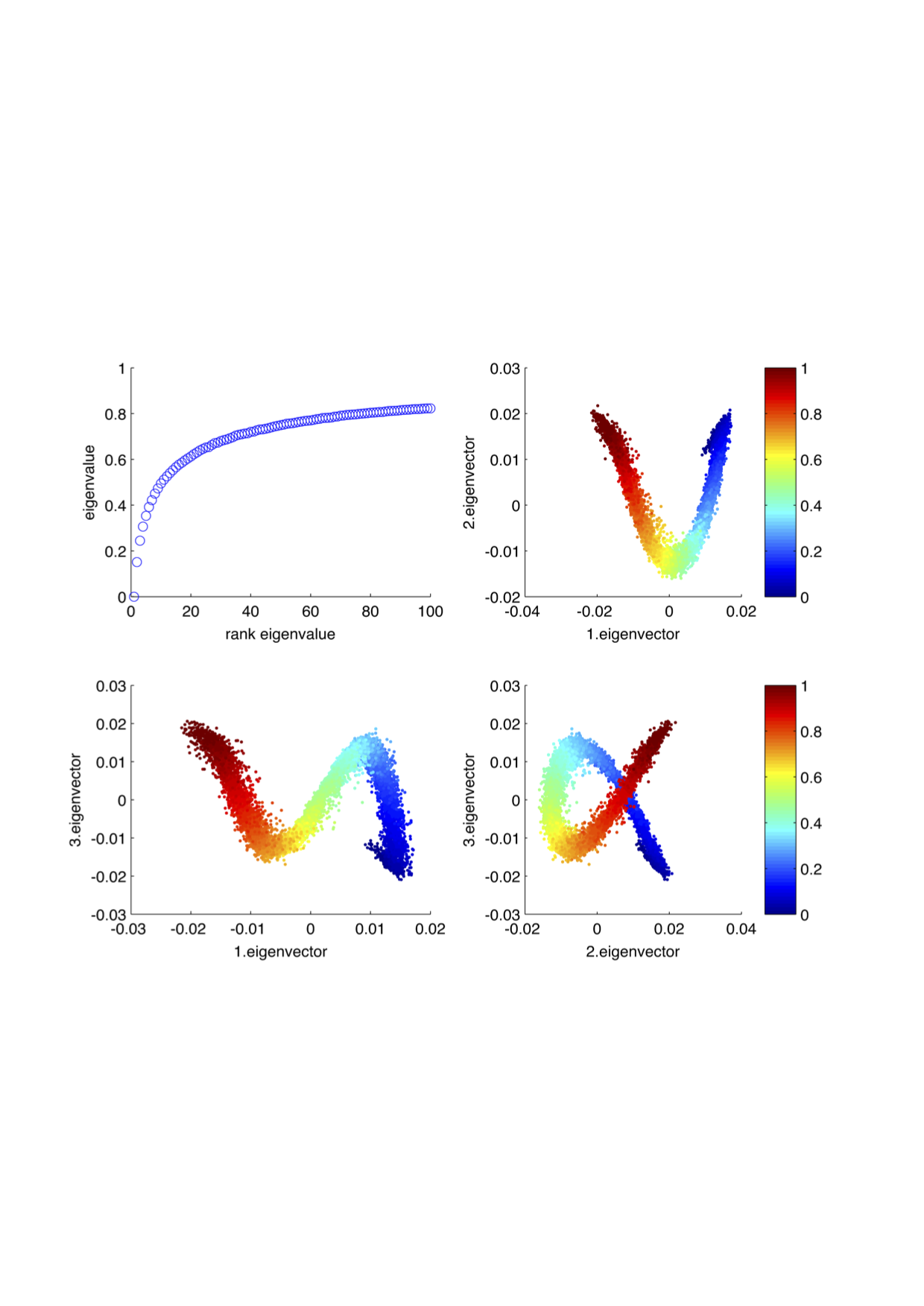

Supplement: S14 Fig — Colorbar indicates the normalized time. Blue dots represent old nodes, whereas red dots are younger nodes in the network. Top panel: spectrum of the normalized laplacian Ln and network projection into the eigenspace spanned by the first and second non-trivial eigenvector of the laplacian matrix Ln. Bottom panel: projection into the eigenspace spanned by the first and third non-trivial eigenvector, respectively, vs. the second and third non-trivial eigenvector of the normalized laplacian matrix Ln. (TIF) [file pone.0156505.s015.tif]

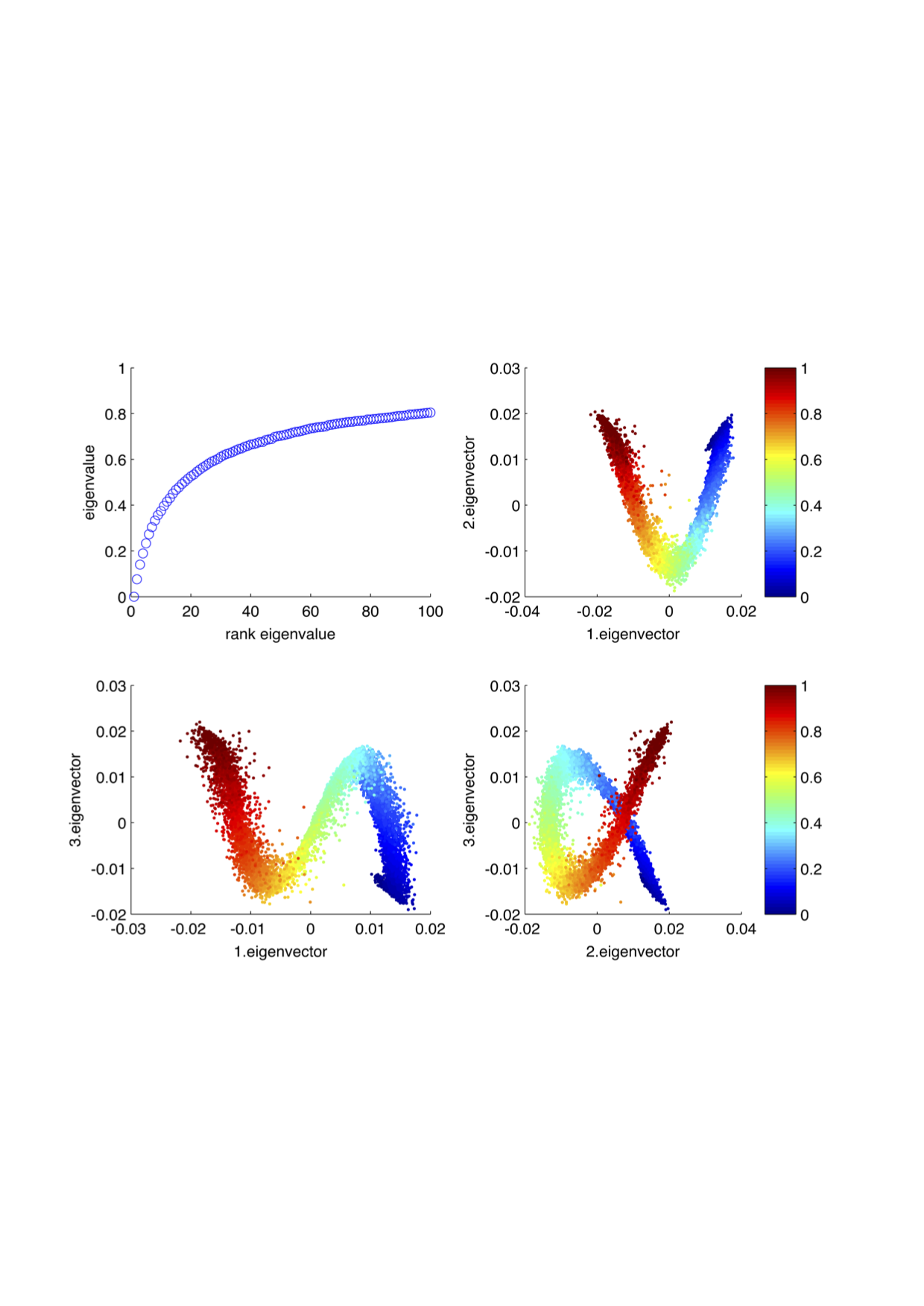

Supplement: S15 Fig — Colorbar indicates the normalized time. Blue dots represent old nodes, whereas red dots are younger nodes in the network. Top panel: spectrum of the normalized laplacian Ln and network projection into the eigenspace spanned by the first and second non-trivial eigenvector of the laplacian matrix Ln. Bottom panel: projection into the eigenspace spanned by the first and third non-trivial eigenvector, respectively, vs. the second and third non-trivial eigenvector of the normalized laplacian matrix Ln. (TIF) [file pone.0156505.s016.tif]

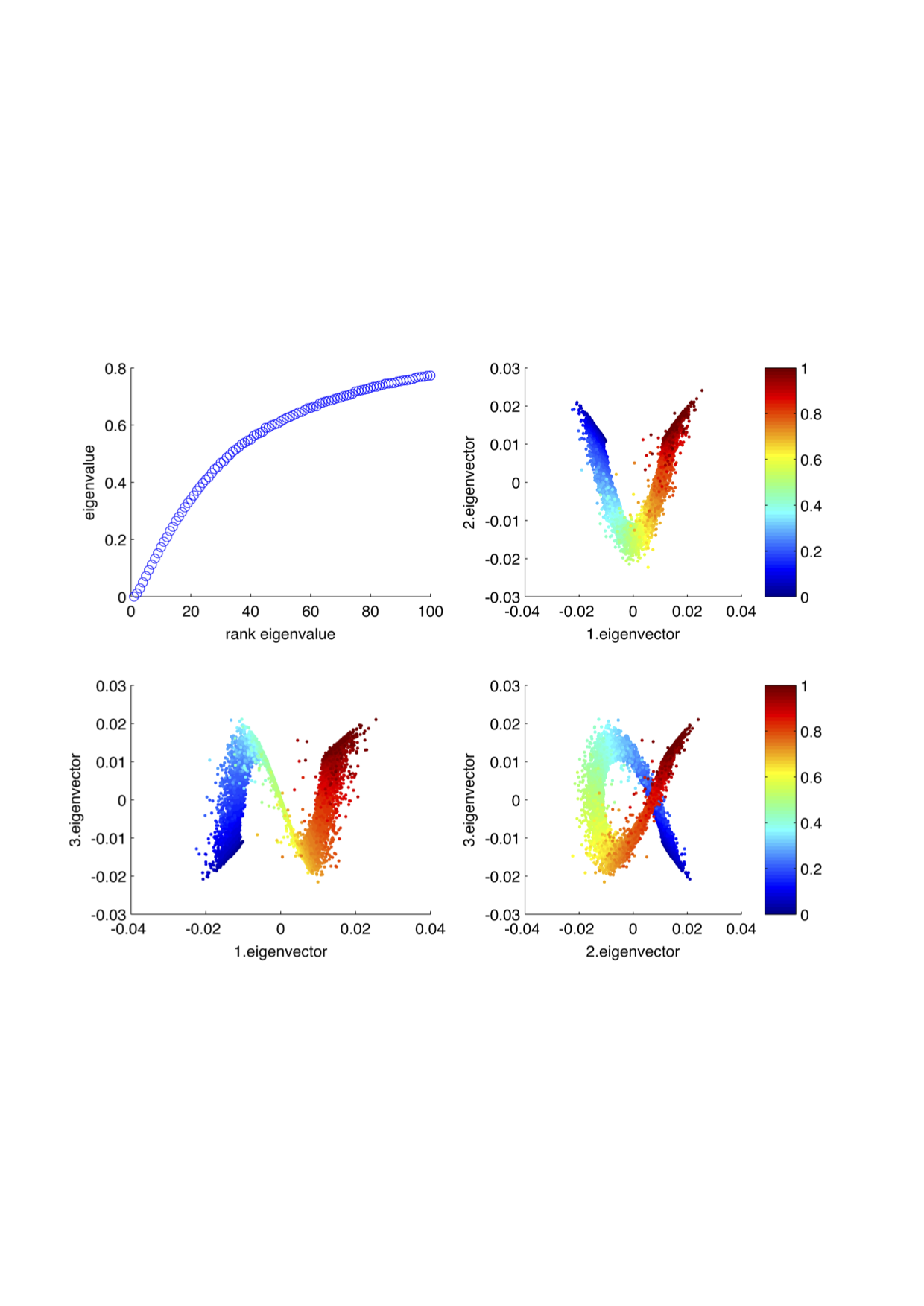

Supplement: S16 Fig — Colorbar indicates the normalized time. Blue dots represent old nodes, whereas red dots are younger nodes in the network. Top panel: spectrum of the normalized laplacian Ln and network projection into the eigenspace spanned by the first and second non-trivial eigenvector of the laplacian matrix Ln. Bottom panel: projection into the eigenspace spanned by the first and third non-trivial eigenvector, respectively, vs. the second and third non-trivial eigenvector of the normalized laplacian matrix Ln. (TIF) [file pone.0156505.s017.tif]

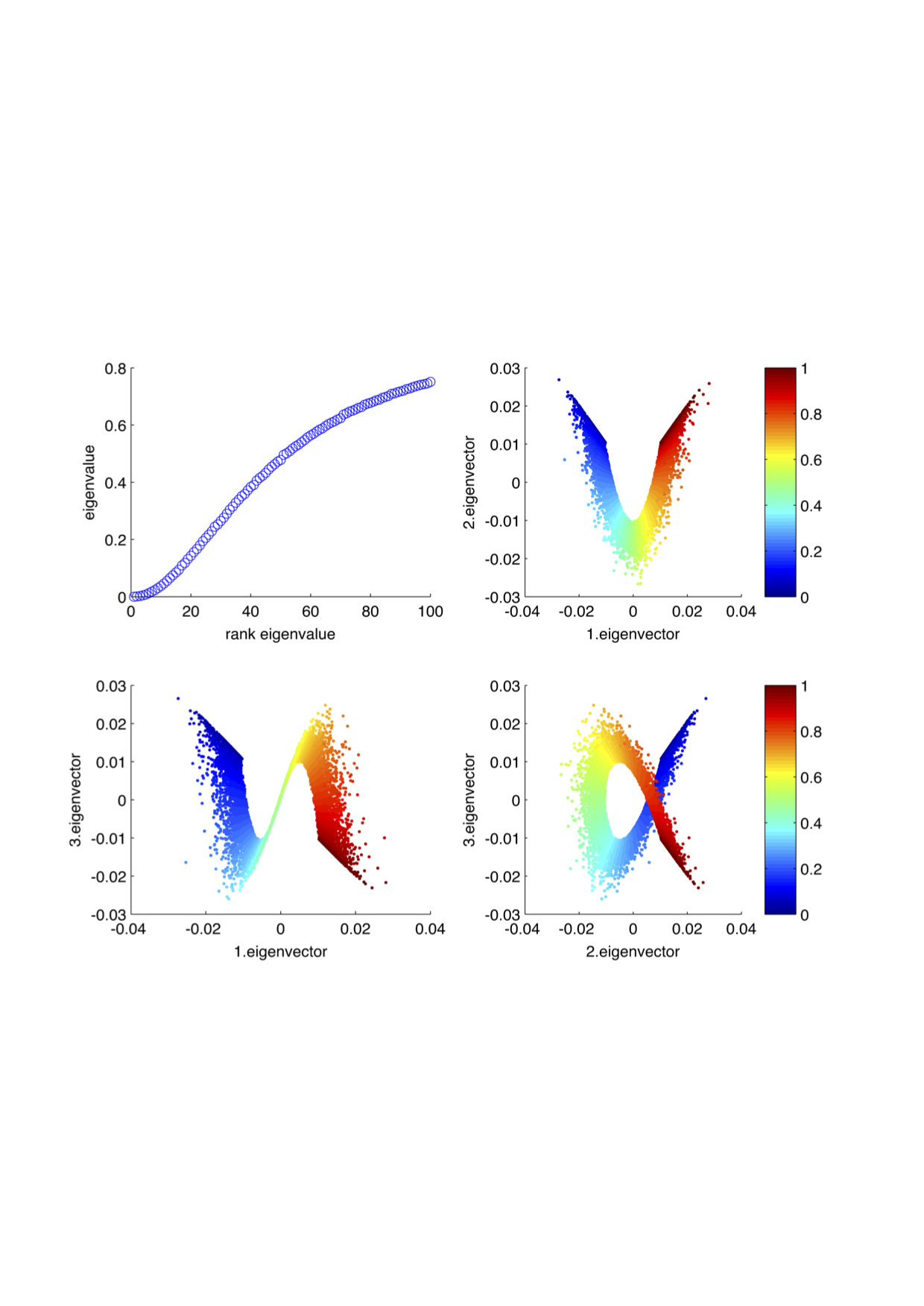

Supplement: S17 Fig — Colorbar indicates the normalized time. Blue dots represent old nodes, whereas red dots are younger nodes in the network. Top panel: spectrum of the normalized laplacian Ln and network projection into the eigenspace spanned by the first and second non-trivial eigenvector of the laplacian matrix Ln. Bottom panel: projection into the eigenspace spanned by the first and third non-trivial eigenvector, respectively, vs. the second and third non-trivial eigenvector of the normalized laplacian matrix Ln. (TIF) [file pone.0156505.s018.tif]

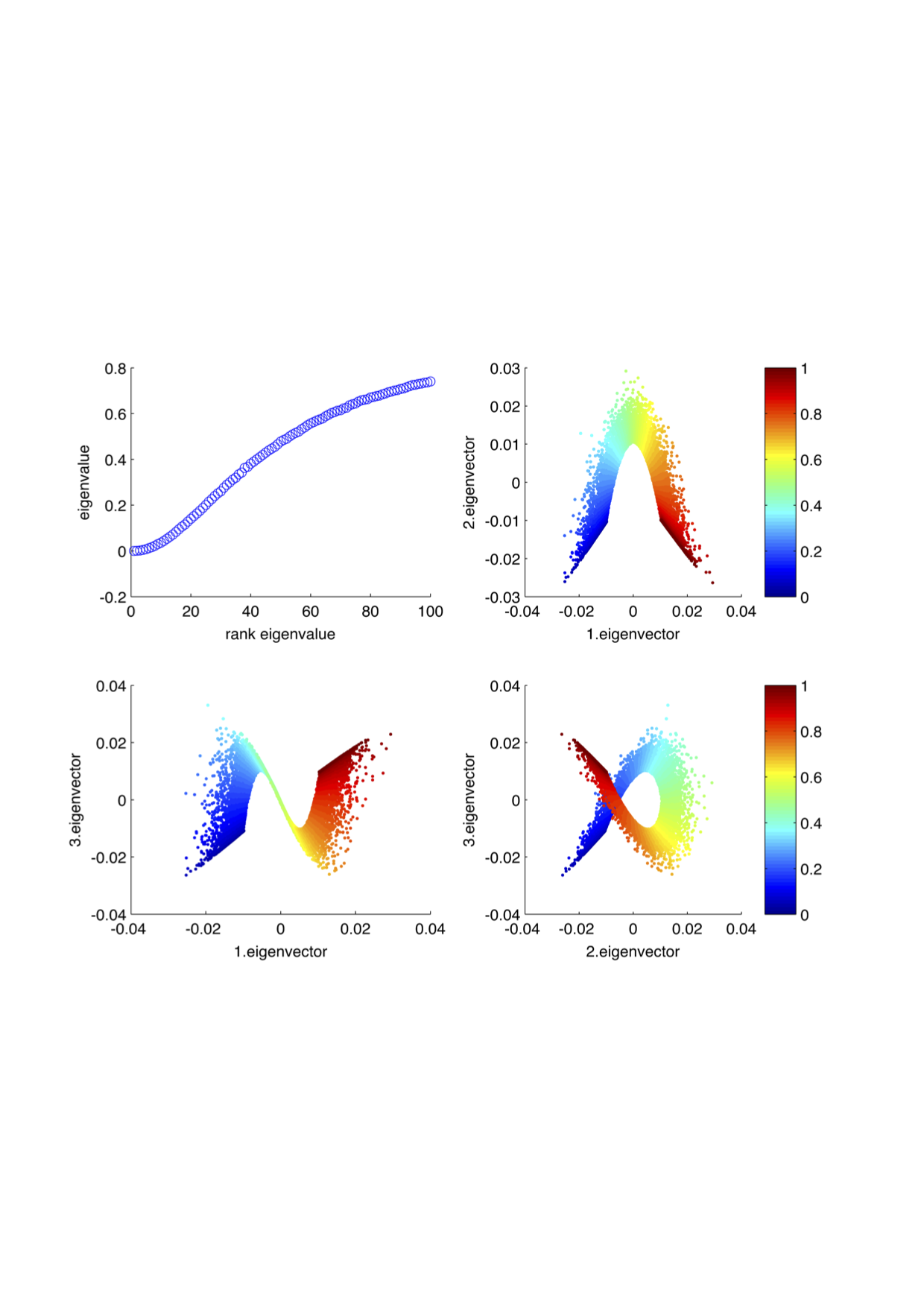

Supplement: S18 Fig — Colorbar indicates the normalized time. Blue dots represent old nodes, whereas red dots are younger nodes in the network. Top panel: spectrum of the normalized laplacian Ln and network projection into the eigenspace spanned by the first and second non-trivial eigenvector of the laplacian matrix Ln. Bottom panel: projection into the eigenspace spanned by the first and third non-trivial eigenvector, respectively, vs. the second and third non-trivial eigenvector of the normalized laplacian matrix Ln. (TIF) [file pone.0156505.s019.tif]
